# Digital fist bumps: searching for datafication and digitalisation in everyday CrossFit coaching practice

**DOI:** 10.3389/fspor.2024.1411176

**Published:** 2024-10-21

**Authors:** Sandra Krugly, Jason Tucker

**Affiliations:** ^1^ Department of Sports Science, Faculty of Learning and Society, Malmö University, Malmö, Sweden; ^2^ Department of Global Political Studies, Faculty of Culture and Society, Malmö University, Malmö, Sweden

**Keywords:** CrossFit, coaching practice, datafication, digital platforms, everyday, digital fitness revolution, rehumanise

## Abstract

The research presented here explores the nuances of data collection and sharing via digital platforms in everyday CrossFit coaching practice. There is a growing body of work on data and digital platforms in CrossFit, though currently there is a lack of understanding of the role of coaches in these processes. Empirically grounding the digital fitness practices of CrossFit coaching is essential for our understanding of the sport, as well as to critically engage with the dominant socio-technical narratives of the digital fitness revolution: narratives that obscure the agency of coaches. This research foregrounds the coaches’ agency and lived experiences, focusing on their everyday coaching practices around data and digital platforms. Six semi-structured in-depth interviews with CrossFit coaches in Sweden were undertaken in 2023. These focused on if, when, how and why they collect, or encourage their participants to collect, data on their training and share this via digital platforms. The findings reveal several different, though interrelated, areas where the CrossFit coaches can be seen as mediating between often competing narratives around data and digital platforms. These everyday practices include mediating between group vs. individual training, data collection and sharing vs. “moving well”, CrossFit's methodology of quantification of fitness vs. the needs of the participants and navigating the techno-solutionist vs. reductionist narratives around digital fitness tracking.

## 1 Introduction

CrossFit has become a global fitness phenomenon over the last two decades. Despite its popularity there is very little research on CrossFit, how it is practiced and why it has expanded so quickly (1). This includes the pivotal role that CrossFit coaches and their practices play in this phenomenon. The growth in the popularity or CrossFit is against the backdrop of the widespread adoption of digital technologies and online platforms in the fitness industry (2). While the impact of the digital fitness revolution varies between sports (3), there has in general been an explosion in the use of personal informatics tools (4), which has resulted in shifting consumer demands on fitness tracking (5, 6). There is increasing pressure on fitness organisations to align with the landscape of these technological innovations (7), pressure which is also felt by coaches. While these technologies can facilitate collaboration between coaches and their clientele (4, 8–10), it also provides new challenges and burdens for coaches (11). The research presented here explores these changes. It does so by focusing on the nuances of data collection and sharing via digital platforms in everyday CrossFit coaching practice.

Nash (12) describes CrossFit coaches as being specialised fitness workers that take on a hybrid role, combining skills required of a sports coach, group training instructor and personal trainer. One of the fundamental ideas that is reinforced, not only in the CrossFit coaches’ education, but also in the workout design, is that the results should be observable, measurable, and repeatable (13). Yet, there is little understanding on if, when, how and why CrossFit coaches collect or encourage their participants^1^ to collect and share data via digital platforms in their everyday coaching practice. Where research does exist, it focuses on digital applications, such as machine learning, to predict and prevent injuries (14), attempts to quantify the multimodal training format of CrossFit (15), how to do so and identify individual training targets (16), or the impact of digital sports on CrossFit athletes during the COVID-19 pandemic (17). The existing literature also addresses aspects of the role of digital spaces and platforms in CrossFit. For example, Dawson (1) explores the culture of CrossFit, seen through the lens of it being a reinventive institution, whereas Pekkanen et al. (18) frames CrossFitters as members of an in-real-life and digital consumer tribe. Edmonds [(19), p. 203], reflects on the geographies of everyday life in a box (the name of CrossFit gyms), and the connection between the local and the global network of CrossFitters through extended digital social relations.

What these studies show is that empirically grounding the digital fitness practices of CrossFit coaching is essential for our understanding of the sport, as well as to be able to critically engage with the dominant socio-technical narratives of the digital fitness revolution narratives which obscure the agency of coaches. To address this, the following question guided this research: What are the everyday practices of data collection, sharing and use of digital platforms in CrossFit coaching?

The research drew on Pink's et al. (20) concept for the need to “rehumanise” research on digital technologies and datafication. This call is a response to what Pink et al. (20) see as two dominant competing narratives: the mythologised techno-solutionism and the counter narrative of the erosion of social relations due to an over reliance on data-driven, and reductionist technologies. To avoid these, they emphasise that the everyday and the mundane is a vital site for research on digital technologies (20). Regarding this research, rehumanisation is useful as it opens the door for a more nuanced exploration of digitalisation and datafication in everyday CrossFit coaching practice.

Digitalisation can be understood as “the way many domains of social life are restructured around digital communication and media infrastructures” [(21), p. 556]. Datafication refers to two processes, “… the transformation of human life into data through processes of quantification, and the generation of different kinds of value from data” [(22), p. 3]. While concept of datafication, and the process it seeks to describe, have been the subject of critique (23–25) it provides a useful conceptual framework to explore the relationship between quantification and value creation.

To empirically ground our understanding of data collection and digital platform use in CrossFit coaching practice, six in-depth semi-structured interviews with qualified CrossFit coaches in Southern Sweden were conducted between February and April 2023. The findings revealed that all the coaches interviewed used data collection, sharing and digital platforms in different ways. This related to the coaches’ various understandings of what their role as a coach was, and where the line should be drawn between the coaches’ and participants’ responsibility for monitoring the participants’ training progression. This was reflected in the differing views on if, when, how and why data collection and sharing data was seen as helpful or harmful to the participants’ motivation and fitness.

## 2 Method

Six in-depth semi-structured interviews were undertaken with CrossFit coaches (who combined worked in nine different boxes) in Southern Sweden between February and April 2023. The four selection criteria were; (1) The interviewees had to be at least CrossFit level one certified coaches.^2^ (2) The interviewees had to regularly coach CrossFit group classes—personal training being outside the scope of this research. (3) They coached in Sweden^3^. (4) Diversity in gender identity, as well as years coaching CrossFit, coaching level and classes coached per week was sought. The interviewees were all identified through a snowball sampling technique.

**Figure 1:**
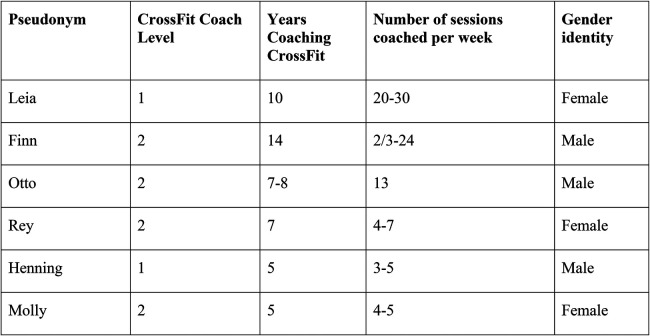
Information on the interviewees.

The interview guide was divided into two parts: the first being on the coaches’ background details (see Figure 1) and the second focusing on their everyday coaching practice related to data and digital platforms.^4^ Key terms were standardized between the authors.^5^ Interviews lasted on average 35 min. The interviews were conducted either face to face or on the phone, in either English or Swedish. Only one of the two authors was present at each interview. Audio recordings were taken, transcribed, with pseudonyms provided for the interviewees and any information that could lead to their identification was removed. Informed consent was secured for all interviewees.

To analyse the data, a multi-stage thematic analysis was undertaken following Braun and Clarke (26, 27). After transcription, the interviews were reviewed independently, and initial themes were identified. Following this the final themes and sub themes were collaboratively developed by both authors, with the data being recoded following this step. The final stage was another review round and the selection of quotes which were representative of broader points of interest. The key themes identified, and the findings related to these, are presented below.

## 3 Results

### 3.1 Not collecting data about the participants

Despite CrossFit's clear methodology of data collection, as found within the coaching training guides (13), the importance the interviewees’ placed on the collection of data from participants in a group training setting varied considerably. This related to their individual coaching styles and whether their focus was mainly on performance, movement quality or scores. As Finn's perspective on what data he encourages his participants to collect reflects:

Not so much on what numbers are on the bars (the weight lifted) or on their times or anything (…) I don't so much collect numbers, but I look at participation in that hour^6^ (…) how invested they are in the hour that I coach, and how they pick up on my instructions on how to perform.

Finn reflected on encouraging participants to share with him how their body felt when they are training. Several other interviewees also noted that they were hesitant to record the participants scores, or encourage them to do so, as they thought it might diminish the importance of movement quality for the participants. This relates to an identifiable tension between prioritising quality of movement and the CrossFit ideology which encourages the measurement of progress. Many of the interviewees noted that navigating this tension was an everyday challenge for them in their coaching practice.

Another factor as to why some of interviewees did not collect data on participants was that they saw data collection as the participants’ responsibility. Some coaches encouraged participants to record their progression for themselves, as it was seen to be in their interest to do so. As Otto explained:

In the role of group exercise instructor, I do not collect any data, (…) No one that works as a coach in the Box works with tracking directly, that is not in the coach's control, but we let people do it if they want.

Rey was more encouraging of participants collecting their data, stating: “(…) we don't collect data, we encourage them to keep it themselves, to write it down somewhere in the phone or in some form of book at each training session (…)”. Leia reflected on why some participants are not interested in collecting data:

I think that if people had more information they would see their development from week to week, and umm, that would be really great for them. But I don't think that many of them are doing it. Maybe after training for a few years or so, but not in the beginning. I think they just show up to the class, they don't want to think so much, they just want to go there, to show up, as it's nice to have someone just tell you what to do.

It was also noted by some of the coaches that due to the nature of CrossFit being group training, it is not possible for the coaches to track all the participants even if they wished to do so to better inform their coaching practice. Collecting data, beyond attendance, was simply not an option as they could not keep track of all the class members simultaneously.

### 3.2 Encouraging the participants to record their data

While none of the coaches themselves recorded data from group training sessions, some provided platforms for participants to record their scores and encouraged them to do so. Both Henning and Molly emphasised the need to identify the participants’ fitness level to better coach and adjust the level (scale) during the session to the individual participant. This was seen to be of the upmost importance when participants were new to CrossFit, new to the coaches or had not previously been taking part in group training sessions at their box. As Henning noted:

The information that I collect is, above all, knowledge relevant to beginners more than about the advanced members. Especially if they are people I have not met before in the Box. The information that I recommend them to register is above all their lifting so that they can see progression, but also for competition and sparring, pep and to push each other a little so that it is visible in the app.

While Henning focused on beginners measuring their progress, Leia's practice was different. Leia believed that it was the advanced participants who benefited most from recording their data. This points to variations in participants competence in the role of data in the coaches’ practice.

The recording of data was also used as an incentive so that the participants were more engaged in the group training session. As Henning noted, if the participants can see what others have previously done on the WOD (Work Out of the Day^7^), then they may think “if they can do it, so can I”.

### 3.3 Offline data collection and sharing with the local CrossFit community

Regarding offline forms of data collection and sharing, four out of six interviewees reflected on the use of whiteboards in their boxes. As Dawson [(1), p. 373] notes “one the central features of any CrossFit box is the whiteboard, which typically broadcasts not only the warm-up and WOD, but also the names and scores of everyone who worked out on a particular day … the whiteboard like the mirror [which are common in other gyms but not in boxes], enables self- and mutual surveillance”.

For the interviewees there was no standard approach to the use of the whiteboards, but their presence in boxes was seen as normal. This was highlighted by the fact that whiteboards were the only medium of offline data sharing that was raised by the participants. Finn explains how the whiteboard is used in his box:

Yes, so what we do every day, we as a coach come in the morning and open up, write down the session warm-up, main part and cool down, that is the whole session, and then next to that on the board they prepare the area of the whiteboard where one can write up ones’ performances. And sometimes it's like that, it goes on like a whole day, up to a 15-hour day, and no one has signed up. And then there are other days when it is full.

For some of the coaches interviewed, the use of whiteboards was not their decision, as Leia noted: “Of course, yeh, he (the owner) sometimes, on the whiteboard, you know, the WOD he would write the times and people's names. For other interviewees however, they introduced their own whiteboard to make data sharing even more accessible to their immediate class. Finn noted how he did so to try to encourage people to ‘dare’ to share their results: ‘I have a small whiteboard on the side so—I’m trying to make it very readily available for them, and make it part of the class, so as to make them aware of their data’”.

The agency of the participants was a common theme for all the coaches when they discussed whiteboards. Some coaches more explicitly encouraged their participants to put up their data, for example Otto and Molly. However, Otto reflected on the role of participants’ choice in sharing data:

Well … you are more or less free to write your result on the whiteboard, but it is not logged (digitally), it is perhaps more to show what results you got, so that others can compare themselves.

When reflecting on the reasons the participants may wish to “write up” their result, being proud of one's achievements, self-monitoring one's training, motivation and encouraging friendly local competition were all raised. As Finn reflected:

Anyone can come into a gym and lift some dumbbells and pull on a machine and then walk out, day in and day out, without actually thinking about what it is they are doing. If we as coaches can motivate them to just write up their times, it's a step closer to understanding their training better.

However, when describing the participants use of whiteboards to share their data, Leia noted:

I think, yeh, it's both and good and bad because there are people who really like the competition, the competitive side of it, but at the same time it's important that you can see the people who just want to go there and have a good time as well.

For others, their box rejected the use of offline public data sharing, as Rey explained:

We have chosen to work on lowering the excitement, as it were, as you shouldn't compare yourself with anyone other than yourself, and therefore we don't have leader boards or scoreboards at the moment.

The target audience for the offline data sharing was, in all but one circumstance, meant for the other box members who came to the box to train. They were always referred to as being in a public and visible place, such as in the entrance way or on the wall of the box.

The distinction between the whiteboard as being an offline data sharing medium was however, blurred. As will be discussed in the following sections, some of this data was shared online as well. For Molly, the whiteboard was seen as an immediate and local way to share data which complemented her participants use of a digital platform (an online app called SugarWod) upon which her box heavily relied:

We can sometimes write a small list on the board with the best scores off the day, because sometimes members do not register their results immediately (on the app) but wait until the evening, and if you notice that there was someone who got a great time, you can write down that time if you want people in the following workouts to push themselves.

### 3.4 Online data collection and sharing not using a CrossFit specific app

In only one case did an interviewee note how the participants’ data was shared publicly through their box website. Here we see the interaction of the offline and the digital forms of data collection and sharing, though the lack of agency of the participants was also reflected upon. As Finn noted:

… No matter how full or how empty it is, we take a picture of the whiteboard and post it on our website under a tab called like “Today's WOD Results”. A rolling database, I don't know how far back, you can check it, but maybe I don't know if this is a data grab: that we take that information and still store it somewhere for anyone to look at afterwards if they want.

When reflecting on the value of this public sharing of data online, Finn noted that it allowed the participants to have a “little competitive spark in their everyday life” and encouraged them to be less introverted, and to establish and maintain relations with their local box community. This ties in with the findings of Dawson (1), Pelkanen et al. (18) and Edmonds (19) on the role of the digital platforms in community development and cohesion in CrossFit.

The use of social media by the participants to share their data was only raised once, and was not encouraged. Leia spoke of how it can have a negative impact on the participants:

I think it's a lot of Instagram as well, a lot of comparing to others and then data has a very specific role, always comparing to others and maybe even though you are not on the same level as them.

This idea of the danger of comparison with others, especially those who are on “another level” was a key theme across for all the interviewees who were cautious about use of whiteboards and digital platforms to share data.

### 3.5 Online data sharing using CrossFit specific apps

Only two out of the six interviewees noted that they encourage the use of CrossFit specific apps to collect and share data as part of their everyday coaching practice. Henning stated how he encouraged some participants to share their data on the SugarWod app to spur on other box members. As he explained:

It is partially shared officially; we have introduced a screen quite recently that sits down there in the entrance that shows today's WOD and those who register their results are seen on the screen with their results.

As such, a digital platform for data sharing is used, but the intention is to make the data available locally. Essentially Henning used the app as a digital local whiteboard in his coaching practice. For Molly, encouraging the participants to use SugarWod and upload as much data as possible was an essential part of her coaching practice. This was in part justified so as to create a supportive online community, through which local box members could contribute to the community via data sharing and recognising each other's achievements. Molly also noted that these apps provided coaches with a lot of data on the individual participants, such as previous coaching notes and injuries. This data could be accessed by Molly on her phone during training sessions to inform her coaching practice. However, Molly also stated that she tells the participants that:

It is not prestigious to register their results, but it is more about helping each other and even when you go in and register your results, you can go in and give each other digital fist bumps. So, everyone who shares their results, you see that they actually go in and fist bump each other, also to de-dramatise it so that not only super good scores are there, but they are mixed.

Both Henning and Molly also raised the issue of agency in what data the participants share on SugarWod and with whom. As Henning reflected: “They can choose whether it should be public or private on their account, so that each member can set it if they want others to be able to see and share their registered results”.

For Finn, while he previously encouraged the use of an app called MyWod, he noted:

I don't know, it's been a long time since I heard that being used. It's not a media we use anymore, I think we don't try to put a lot of value in the data collection in that way, so it's a no. I don't think there is anything (in my box) like that anymore.

In Otto's case, it was not that the app fell out of favour, but that his box coaches and management rejected their use. Regarding online or digital software to collect and share data, Otto noted: “There has never been a serious discussion during my time at the Box about whether to use such a service”. He claimed that the reason for this rejection was that the apps were too comprehensive, making them less appealing as many existing systems would need to be replaced to use the app. Further to this, regarding the value added of such apps, Finn noted:

I think the people who run the gyms (boxes) feel that it is a big cost for such a service and that they are a little skeptical about the value in it. Maybe not necessarily the value in logging your training, but the value in having it happen in that way (on an app) vs. people writing their results on the whiteboard, or them writing it in their own mobile phone.

## 4 Discussion

This research sought to shed light on the use of data and digital platforms in CrossFit coaches everyday coaching practice. Inspired by Pink et al.'s (20) concept of rehumanisation, the findings speak to several different, though interrelated, areas where the CrossFit coaches can be seen as mediating between often competing narratives around data and digital platforms. These include reconciling tensions between group vs. individual training, data collection and sharing vs. “moving well”, CrossFit's methodology of quantification of fitness vs. the needs of the participants and navigating the techno-solutionist vs. reductionist narratives around digital fitness tracking.

This research can be understood as adding another layer to the complex hybrid role identified by Nash (12) that CrossFit coaches have to adopt. The findings reveal the limited value, and perceived risks, many interviewees placed on the use of data collection and sharing via online and offline platforms in their everyday coaching practice. For those interviewed, the digital fitness revolution has had limited impact on their coaching practice, aligning with Hirsh's (3) claim of its varied impact across sports. The interviewees’ practices also seem to push back against techno-solutionist narratives which posit technology as the best and only means to solve problems such as injury prediction and prevention (14), quantify the multimodal training format (15), or the personalisation of training targets (16).

The coaches who encouraged the use of digital platforms, and the sharing of participants’ data (locally), only did so to monitor individuals in a larger group setting and to provide individual feedback, as noted by Loia and Orciuoli (11) with regard to to role of ITC in large group sessions in sports in general. Surprisingly, the coaches did not discuss how the broader CrossFit community, beyond the local box, played a role in their coaching practice. This localisation contributes to our understanding of the role of space and place and the digital CrossFit community, as seen in the work of Dawson (1), Pelkanen et al. (18) and Edmonds (19).

This research was exploratory in nature, and thus limited in scale, scope, and theoretical ramifications. The aim was to address the lack of research on CrossFit coaching practice and to establish a means to meet the challenge that the narratives of the digital fitness revolution often obscure the agency of coaches. A broad and flexible approach was adopted to scope the field and best identify the most pertinent areas upon which to focus future research. The most notable of these is research that explores the psychological perspectives on the use of data and digital platform in CrossFit coaching. In addition, there is a need to engage with CrossFit coaches and support their hybrid role in navigating the use of data and digital platforms. The coaches’ perspective, given their epistemic role in the community, is also a valuable one to explore more meaningful data collection and sharing approaches in CrossFit. Overall, it is hoped that this piece can serves as a catalyst for research that takes the empirical and the everyday as the jumping off point for better understanding the role of data and digitalisation in CrossFit coaching and coaching practice in general.

## Data Availability

The research data can be made available upon request to the authors.

## Footnotes

^1^We use the term ‘participants’ here in reference to individuals who are CrossFit members and attending the CrossFit classes. Those who we interviewed for the research are referred to as ‘interviewees’.

^2^This was important as the inclusive nature of CrossFit means that a range of people can coach classes and provide one to one training as self-identified CrossFit coaches, even when they have no formal CrossFit training or certification. The research was aimed specifically at those who were officially recognised CrossFit coaches, given their epistemic authority in the community.

^3^Due to the various levels of restrictions and lockdowns of boxes during the COVID-19 pandemic, between and within countries, for this piece we limited the study to the Swedish context. In Sweden the boxes remained open during the pandemic and were largely unaffected by the restrictions. In addition, there is a lack of research on CrossFit in Sweden.

^4^The five questions covered; 1) The data they collect or encourage their clients to collect about their training. 2) The practices surrounding the dissemination of this data. 3) The role of online and in-real-life sharing of data. 4) The role of data collection in their coaching practice. 5) The role of data sharing in their coaching practice. A range of follow up and probing questions were also used, but these varied given the semi-structured interview design.

^5^The interview guide clarified the definition of data in the context of CrossFit: “Data about individuals’ performance during group training, such as, rep counts on AMRAPs or time on RFTs. In general, WOD information”. This was also in the information letter shared with the participants before the interview. Examples of digital platforms that we could provide included SugarWOD, CrossFit.com and social media platforms etc. However, these were only provided upon request.

^6^This is the standard duration of a CrossFit class.

^7^The WOD is decided centrally by CrossFit HQ and all boxes globally follow this training plan.
